# *Chlamydia trachomatis* Genotypes and the Swedish New Variant among Urogenital *Chlamydia trachomatis* Strains in Finland

**DOI:** 10.1155/2011/481890

**Published:** 2011-05-18

**Authors:** Suvi Niemi, Eija Hiltunen-Back, Mirja Puolakkainen

**Affiliations:** ^1^Haartman Institute, Department of Virology, University of Helsinki, P.O. Box 21, 00014 University of Helsinki, Finland; ^2^Clinic of Venereal Diseases, Skin and Allergy Hospital, Helsinki University Central Hospital, P.O. Box 160, 00029 HUS, Finland; ^3^Department of Infectious Disease Surveillance and Control, National Institute of Health and Welfare, P.O. Box 30, 00271 Helsinki, Finland; ^4^Helsinki University Central Hospital, Laboratory Division (HUSLAB), Department of Virology and Immunology, P.O. Box 400, 00029 HUS, Finland

## Abstract

Our aims were to genotype *Chlamydia trachomatis* strains present in urogenital samples and to investigate the occurrence of the Swedish new variant of *C. trachomatis* in Finland. We genotyped 160 *C. trachomatis* positive samples with *ompA* real-time PCR and analyzed 495 samples for the new variant. The three most prevalent genotypes were E (40%), F (28%), and G (13%). Only two specimens containing bacteria with the variant plasmid were detected. It seems that in Finland the percentage of infections due to genotypes F and G has slightly increased during the last 20 years. Genotypes E and G appear to be more common, and genotypes J/Ja and I/Ia appear to be less common in Europe than in the USA. Although the genotype E was the most common genotype among *C. trachomatis* strains, the new variant was rarely found in Finland.

## 1. Introduction

*Chlamydia trachomatis*, an obligate intracellular bacterium, is worldwide a common cause of sexually transmitted infections. Even asymptomatic infections can lead to serious sequelae such as infertility and ectopic pregnancy. The infections are very prevalent among adolescents, and reinfections are common [1]. Young people with *C. trachomatis* constitute an important target group for public health interventions. Since 1995, the number of *C. trachomatis* infections has been officially notified in Finland, and the number of notifications has been increasing. Lately, there has been around 14 000 notified cases annually (250 cases/100 000 inhabitants) [2]. In Finland, *C. trachomatis* infections are diagnosed mainly with sensitive and specific nucleic acid amplification tests (NAATs), but these tests do not differentiate between genotypes. 

The seroimmunological analysis of *C. trachomatis* major outer membrane protein (MOMP) first leads to the identification of ≥15 different serovars, and sequence differences in the* ompA* gene, especially in the areas coding the variable domains of MOMP, later confirmed this discrimination [3, 4].* C. trachomatis *types A–C cause trachoma, types D–K cause urogenital infections, and types L1–L3 cause lymphogranuloma venereum (LGV). *C. trachomatis* has also a cryptic plasmid which is commonly used as a target sequence in diagnostic NAATs. In 2006, there was an unexpected fall in *C. trachomatis* cases in Sweden, which was caused by the appearance of a new variant of *C. trachomatis* (nvCT) with a 377 base pair deletion in the cryptic plasmid [5]. At the same time, the proportion of urogenital samples that tested positive by Cobas TaqMan CT Test, old version (Roche), decreased slightly in Southern Finland (Dr. Jukka Suni, HUSLAB; personal communication).

The purpose of this work was to set up a real-time PCR-based method for genotyping *C. trachomatis* (types D–K and L1–L3) in urogenital samples. Additionally, we wanted to set up a method for detection of the Swedish nvCT and to study the occurrence of this variant in Finland. 

## 2. Materials and Methods

### 2.1. Clinical Samples and DNA Extraction

In 2008, 160 unselected *C. trachomatis* positive specimens from females and males were collected for genotyping. The first-void urines (*N* = 82), cervical/vaginal swabs (*N* = 75), and conjunctival swabs (*N* = 3) had been sent to HUSLAB, Department of Virology for *C. trachomatis* nucleic acid testing. HUSLAB, the diagnostic laboratory of the Helsinki University Central Hospital serving the capital area, used at that time both Aptima Combo 2 Assay (Gen-Probe) and Cobas TaqMan CT Test (Roche). Of the 160 samples, 84 tested *C. trachomatis* positive with Gen-Probe and 76 with Roche test. Additionally, 38 *C. trachomatis* negative clinical samples were included as negative controls. To estimate the prevalence of the Swedish nvCT in Finland, 495 urogenital samples were studied. Of these samples, 469 were *C. trachomatis* positive with Aptima Combo 2 Assay (Gen-Probe) (*N* = 414) or Cobas TaqMan CT Test, new version (Roche) (*N* = 55), and 26 were negative with Cobas TaqMan CT Test, old version (Roche). As the prevalence of the nvCT at that time was quite high in Sweden [6], we initially planned to collect specimens that tested *C. trachomatis* negative by the old version of the Cobas TaqMan CT Test (Roche), which fails to detect them. However, the test was soon replaced by a new dual-target version by the manufacturer, and the majority of our material consisted of *C. trachomatis* positive specimens.

For genotyping, DNA was extracted from a specimen volume of 400 *μ*L with MagNA Pure Compact instrument (Roche) using MagNA Pure Compact Nucleic Acid Isolation Kit I (Roche) with DNA Bacteria protocol and eluted in 50 *μ*L. For detection of the nvCT, DNA was extracted from a specimen volume of 200 *μ*L with MagNA Pure LC (Roche) instrument using MagNA Pure LC DNA Isolation Kit I (Roche) with DNA I Blood Cells High Performance protocol and eluted in 100 *μ*L. The concentration of total DNA was measured with a NanoDrop 1000 spectrophotometer (Thermo Scientific). DNA extracted from the samples was stored at −70°C until analysis.

### 2.2. Real-Time PCR

Genotyping was performed according to a method described previously by Jalal et al. [7]. The method is based on the sequence variation of the *ompA* gene, and it has two primer sets and eleven genotype-specific TaqMan probes for types D–K and L1–L3. In this study, we used the non-nested version of the method. As controls, DNA extracted from *C. trachomatis* reference strains (types A–K and L2) originally from ATCC (American Type Culture Collection) propagated in McCoy cells (mouse fibroblast cells) [8] was used. 

To screen for the Swedish nvCT, we used a method developed by Catsburg et al. [9]. This method has primers and a TaqMan probe flanking the deletion sequence in the cryptic plasmid of *C. trachomatis*. A clinical sample containing the nvCT, kindly provided by Professor Björn Herrmann, Uppsala, Sweden, was used to construct a positive control for the assay. The sequence flanking the deletion was amplified and cloned into a plasmid vector with Zero Blunt TOPO PCR Cloning Kit (Invitrogen Life Technologies) according to the manufacturer's instructions. Plasmids were extracted with QIAprep Spin Miniprep Kit (QIAGEN), and the DNA concentration was measured with a NanoDrop 1000 spectrophotometer (Thermo Scientific).

Real-time PCR analyses were performed with an ABI 7500 instrument and Sequence Detection Software version 1.3.1 (Applied Biosystems). The primers and probes used in this study were purchased from Applied Biosystems, Metabion International AG, or TAG Copenhagen A/S. The PCR reactions were performed in a 25 *μ*L volume containing 250 nM primers, 100 nM probes, and either 12.5 *μ*L Platinum Quantitative PCR SuperMix-UDG (Invitrogen Life Technologies) or 12.5 *μ*L TaqMan Universal Master Mix (Applied Biosystems). Thermal cycling conditions were, 50°C 2 minutes, 95°C 10 minutes and 40 cycles of 95°C 15 seconds and 60°C 1 minute. Template volume was 2 *μ*L or 5 *μ*L for genotyping and 2 *μ*L from two different samples pooled together for detection of the nvCT.

## 3. Results

Of the 160 *C. trachomatis* positive clinical samples analyzed, 144 (90%) could be genotyped. Ninety % of both first-void urines and swab samples sent for NAAT contained enough chlamydial DNA for the *ompA* PCR. Sixteen samples (10%) remained without a genotype with the non-nested version of the used method which was most likely due to the low amount of chlamydial DNA present in the specimens. The *ompA* genotype distribution of the strains is presented in Tables 1 and 2. Genotypes E (*N* = 57, 40%), F (*N* = 41, 28%), and G (*N* = 19, 13%) were the most prevalent in this study and comprised together over 80% of the *C. trachomatis* findings. The 16 samples that remained negative for genotypes D–K were analyzed for genotypes L1–L3. We did not detect any genotypes L1–L3 in these urogenital samples. All the 38 *C. trachomatis* negative clinical samples tested also negative in genotyping PCR.

Among the 469 *C. trachomatis* positive clinical samples screened for the Swedish nvCT, samples from two female patients were found to contain a bacteria with the variant plasmid (0.4%). One of the samples was first-void urine and the other a vaginal swab. Both of the nvCT were of genotype E, as has been described also in Sweden and elsewhere [6, 21]. Both also harbored a cryptic plasmid with a deletion site in the ORF1, and the sequence flanking the deletion in both strains was identical to that of the Swedish nvCT (confirmed by sequencing) [5]. The clinical samples containing the nvCT were both initially tested by the Aptima Combo 2 Assay (Gen-Probe) that is based on detection of the ribosomal RNA and is thus able to detect also this type of variant *C. trachomatis*.

## 4. Discussion

Typing of* C. trachomatis* strains can be used in epidemiologic surveillance locally or internationally, to assess the changes in genotype distribution and to reveal transmission patterns in sexual networks. It potentially also assists in understanding the pathogenesis of these infections for example, whether genotypes are associated with clinical phenotypes [22] or clearance of infection [20] or differentiating between new, repeated, and persistent infection [23, 24]. This information is elementary in efforts to monitor and prevent spread of *C. trachomatis* infections. Typing of *C. trachomatis* has traditionally been based on differences in MOMP or the *ompA* gene coding it. Lately, also high-resolution genotyping for *C. trachomatis* [24, 25] has been developed. These novel methods could be advantageous especially when strains from a geographically localized area or from a population where certain *ompA* genotypes dominate are analyzed. 

Of the unselected *C. trachomatis* positive specimens sent to the diagnostic laboratory for NAAT, 90% contained enough DNA to allow genotyping. Based on an *ompA* PCR, the most prevalent *C. trachomatis* genotypes in Finland were E, F, and G comprising approximately 80% of the strains that could be typed. The proportion of types F and G seems to have increased whereas the proportion of types D and E seems to have remained rather stable, when set against the typing results from 1987 (monoclonal antibodies) [10] and 1996 (*ompA* PCR and restriction fragment length polymorphism, RFLP) [11]. Although the different methods used in these studies and the relatively small numbers of strains typed could partly explain the observed differences, these might be true changes. Indeed, also in Seattle, Washington, with a method based on monoclonal antibodies, a similar change was reported: the proportion of infections due to serovars F and G increased in 1988–1996 [17]. 

When the type distribution in Europe (Finland, Sweden before the appearance of the nvCT [12, 13], the Netherlands [14, 15], and Portugal [16]) and in the USA [17–20] was evaluated, the proportion of types E and G tended to be slightly lower in the USA than in Europe, while the proportion of types J/Ja and I/Ia seemed somewhat higher in the USA than in Europe. Again, the typing methods varied (see Table 2). However, when the European data is compared to that obtained with *ompA* sequencing in the USA, the aforementioned observations still remain [19, 20]. 

Although the typing method used also detects genotypes L1–L3, we did not detect any L types among the urogenital specimens tested. Classic LGV infections caused by genotypes L1–L3 have been uncommon in the Western Countries, including Finland. However, lately outbreaks of proctitis caused by type L2 among HIV-positive MSM have been reported in many European countries [26]. 

Certain *C. trachomatis* sero-/genotypes have been linked to certain clinical features for example abdominal pain, pelvic inflammatory disease, and pregnancy complications, but also to asymptomatic infections [18, 22, 27–29]. As the associations remain weak at best, it is not likely that typing *ompA* or *ompA* encoded protein MOMP of the urogenital strains will reveal clues to their pathogenicity. Classification relying on other, yet unidentified virulence attributes might better reflect associations of certain *C. trachomatis* subgroups with disease severity, persistence, or asymptomaticity [30]. Also host factors are likely to modulate the outcome of the infection.

The emergence of the Swedish nvCT has clearly pointed out the importance of epidemiologic surveillance and attentive evaluation of results obtained using diagnostic test kits. Diagnostic tests should target sequences that are conservative and essential for the bacterium. As the cryptic plasmid is not absolutely essential for survival of *C. trachomatis*, mutations in the plasmid genes can even lead to a selection advantage for *C. trachomatis* [31]. In this study, we were able to find only two clinical samples with the Swedish nvCT, which was expected: only few cases outside Sweden have been reported and most often the cases were linked to Sweden [32]. Unfortunately, we could not obtain information on our patients whether they had contacts to Sweden because they were both lost from follow-up. Although the decrease in the positivity rate by the Cobas TaqMan CT Test, old version (Roche), in a major Finnish diagnostic laboratory could not be explained by the appearance of nvCT, this exercise has considerably improved our ability to detect novel variants.

## 5. Conclusions

In this study, we showed that the most prevalent genotype in Finland was E (40%). However, the Swedish variant was rare in Finland despite our close proximity to Sweden and the frequent occurrence of genotype E.

##  Conflict of Interests 

The authors declare that they have no conflict of interests.

## Figures and Tables

**Table 1:** The *ompA* genotype distribution (%) of 144 clinical *C. trachomatis* strains from this study (2008) compared with the distribution of types in isolates from the same laboratory in 1996 (*ompA* PCR and restriction fragment length polymorphism, RFLP) and in 1987 (micro-immunofluorescence, MIF) in Finland.

Sero-/genotype	1987 (Saikku & Wang) [10] *N* = 51	1996 (Penttilä et al.) [11] *N* = 122	2008 (This material) *N* = 144
			
B	2	2	ND
E	25	33	40
D	6	9	8
ED	25	ND	ND
D/B	ND	2	ND

total	58	46	48

F	10	22	28
G	4	13	13
K	8	2	5

total	22	37	46

C	0	ND	ND
C/J	ND	6	ND
C-group	ND	8	ND
H	6	3	3
I	6	ND	0
J/Ja	8	ND	2

total	20	17	5

ND: not determined

C-group: genotypes A, C, H, I, Ia, J, K, and L3.

**Table 2:** The *ompA* genotype distribution (%) of 144 clinical *C. trachomatis* strains in Finland (this study) compared with distribution of types in *C. trachomatis* strains in Sweden, the Netherlands, Portugal and the USA.

Sero/ genotype	Finland (this material) *N* = 144* ompA* PCR & probes	Sweden [12] *N* = 237* ompA* sequencing	Sweden [13] *N* = 678* ompA * sequencing	The Netherlands [14] *N* = 438* ompA* PCR & RFLP	The Netherlands [15] *N* = 407* ompA* PCR & RFLP	Portugal [16] *N* = 795* ompA* sequencing	The USA [17] *N* = 11 454 serotyping	The USA* [18] *N* = 480/700 serotyping	The USA [19] *N* = 507* ompA* sequencing	The USA [20] *N* = 102* OmpA * sequencing
B/Ba	ND	0,4	1	ND	1	0,4	2	1	1	ND
E	40	47	39	40	33	40	32	30/33	30	28
D/Da/D-	8	14	9	12	13	13	16	17/19	14	23

total	48	61,4	49	52	47	53,4	50	48/53	45	51

										
F	28	17	21	21	23	17	18	20/17	19	11
G/Ga	13	3	11	8	9	11	2	3/4	4	1
K	5	9	9	4	2	1	4	3/5	5	1

total	46	29	41	33	34	29	24	26/26	28	13

C	ND	ND	ND	ND	ND	0,4	ND	ND	0	ND
H	3	3	2	4	8	3	3	2	1	3
I/Ia	0	3	1	4	7	6	10	12/9	14	15
J/Ja	2	4	7	4	3	7	11	13/10	12	19

total	5	10	10	12	18	16,4	24	27/21	27	37

RFLP: restriction fragment length polymorphism.

*Genotypes of women and men separately.

ND: not determined.
